# Vitamin D in inflammatory diseases

**DOI:** 10.3389/fphys.2014.00244

**Published:** 2014-07-02

**Authors:** Thea K. Wöbke, Bernd L. Sorg, Dieter Steinhilber

**Affiliations:** Institute of Pharmaceutical Chemistry, Goethe University FrankfurtFrankfurt, Germany

**Keywords:** 1α,25(OH)_2_D_3_, VDR, cyclooxygenase, NFκB, NFAT, MKP1, interleukins, innate immune system

## Abstract

Changes in vitamin D serum levels have been associated with inflammatory diseases, such as inflammatory bowel disease (IBD), rheumatoid arthritis, systemic lupus erythematosus, multiple sclerosis (MS), atherosclerosis, or asthma. Genome- and transcriptome-wide studies indicate that vitamin D signaling modulates many inflammatory responses on several levels. This includes (i) the regulation of the expression of genes which generate pro-inflammatory mediators, such as cyclooxygenases or 5-lipoxygenase, (ii) the interference with transcription factors, such as NF-κB, which regulate the expression of inflammatory genes and (iii) the activation of signaling cascades, such as MAP kinases which mediate inflammatory responses. Vitamin D targets various tissues and cell types, a number of which belong to the immune system, such as monocytes/macrophages, dendritic cells (DCs) as well as B- and T cells, leading to individual responses of each cell type. One hallmark of these specific vitamin D effects is the cell-type specific regulation of genes involved in the regulation of inflammatory processes and the interplay between vitamin D signaling and other signaling cascades involved in inflammation. An important task in the near future will be the elucidation of the regulatory mechanisms that are involved in the regulation of inflammatory responses by vitamin D on the molecular level by the use of techniques such as chromatin immunoprecipitation (ChIP), ChIP-seq, and FAIRE-seq.

## Introduction: 1α,25(OH)_2_D_3_ and inflammatory diseases

It is now well established that the physiological importance of the vitamin D status extends far beyond the regulation of bone metabolism. According to its manifold functions in immune homeostasis, increasing evidence relates serum vitamin D levels as well as polymorphisms in enzymes involved in vitamin D metabolism to the incidence of chronic inflammatory diseases like asthma, atherosclerosis and autoimmune diseases (Stojanovic et al., 2011; Summerday et al., 2012; Szekely and Pataki, 2012). However, whether vitamin D exerts a salutatory or deteriorating role in such diseases is still under debate. This review will focus on the knowledge regarding the role of vitamin D in inflammatory diseases by the examples of asthma, atherosclerosis and autoimmune diseases.

## 1α,25(OH)_2_D_3_ and asthma

According to the World Health Organization (WHO), asthma is the most common chronic disease among children (http://www.who.int/mediacentre/factsheets/fs307/en/index.html). In this context, several studies addressed the interrelationship of the maternal as well as infant vitamin D status and the prevalence and severity of asthma. Three studies by Brehm et al. analyzed the relationship between vitamin D levels and asthma severity in Costa Rican, North American and Puerto Rican children, respectively (Brehm et al., 2009, 2010, 2012). Collectively, they found high prevalences of vitamin D insufficiency in asthmatic children and vitamin D insufficiency was correlated with severe asthma exacerbations. However, the prevalence of vitamin D insufficiency was high in Puerto Rican children irrespective of the indisposition from asthma, with roughly comparable percentages between asthma patients and otherwise healthy children (Brehm et al., 2012). Although few studies showed no correlation between serum vitamin D levels and the presence of asthma (Menon et al., 2012; Gergen et al., 2013), many studies state a higher prevalence of vitamin D deficiency in asthmatic children (Freishtat et al., 2010; Chinellato et al., 2011a,b; Ehlayel et al., 2011; Hollams et al., 2011; Bener et al., 2012; Krobtrakulchai et al., 2013) and adults (Li et al., 2011b). Additionally, in many cases a relation between low vitamin D levels and reduced asthma control is found. Furthermore, metabolomic analysis of breath condensates revealed reduced levels of vitamin D metabolites in children with asthma (Carraro et al., 2013). Similarly, enhanced vitamin D binding protein levels were found in bronchoalveolar lavage fluid of asthmatic children (Gupta et al., 2012b). Interestingly, one study describes an age-dependent association between serum vitamin D level and asthma prevalence in children (Van Oeffelen et al., 2011).

A different relationship between the vitamin D status and asthma has been brought up by a northern Finland birth cohort study, which revealed an increased risk of asthma in adults who received high dose vitamin D supplementation in their childhood (Hypponen et al., 2004). In accordance with these findings, a prospective study by Tolppanen et al. revealed an increased risk of wheezing in association with higher vitamin D levels, but no correlation of lower vitamin D levels to respiratory sicknesses (Tolppanen et al., 2013). Another study reinforces the finding of increased susceptibility to asthma after vitamin D supplementation, yet only regarding supplementation of water soluble formulations and not in connection with vitamin D supplementation in peanut oil (Kull et al., 2006).

There is debate as to whether maternal vitamin D levels during the pregnancy influence the susceptibility to asthma of the progeny. Whereas some reports showed no correlation between maternal or cord blood vitamin D levels and an increased risk of childhood asthma (Camargo et al., 2011; Rothers et al., 2011; Morales et al., 2012; Pike et al., 2012; Magnus et al., 2013), another report indicates that high maternal vitamin D levels correlate with enhanced probability of asthma development in children (Gale et al., 2008). In contrast, some reports associate higher vitamin D intake during pregnancy with reduced risk of childhood wheezing and asthma (Camargo et al., 2007; Devereux et al., 2007; Erkkola et al., 2009).

Mechanistically, vitamin D induced protection against airway inflammation has been related to a modulated T cell response to allergens as well as induction of the immunoglobulin-like anti-inflammatory cell surface protein CD200 on T cells, that acts on target immune cells which express the CD200 receptor (CD200R) (Dimeloe et al., 2012; Gorman et al., 2012; Urry et al., 2012). Many authors suggest that the beneficial effect of sufficient vitamin D levels on asthma development results from the immune enhancing effect of vitamin D and the simultaneous prevention of respiratory infections (Ginde et al., 2009; Camargo et al., 2011; Majak et al., 2011; Morales et al., 2012).

Furthermore, there is evidence that the serum vitamin D level has also an influence on asthma therapy, as vitamin D has been demonstrated to enhance glucocorticoid (GC) action and lower serum vitamin D levels are associated with higher corticosteroid requirement, at least in children, or even therapy-resistance (Searing et al., 2010; Goleva et al., 2012; Gupta et al., 2012a; Wu et al., 2012). Additionally, the therapeutic effect of specific allergen immunotherapy has been correlated to serum vitamin D levels (Majak et al., 2012).

Besides serum vitamin D levels also polymorphisms of genes of the vitamin D pathway such as the vitamin D receptor (VDR) have been associated with asthma (Poon et al., 2004; Raby et al., 2004; Saadi et al., 2009; Li et al., 2011a; Pillai et al., 2011; Maalmi et al., 2013), yet, not all studies revealed a correlation between vitamin D pathway polymorphisms and asthma prevalence (Vollmert et al., 2004; Fang et al., 2009).

## 1α,25(OH)_2_D_3_ and atherosclerosis

Another chronic inflammatory disease that is more prevalent in the elderly population is atherosclerosis. Early studies on atherosclerosis development in several animal models revealed an accelerating effect of high doses of vitamin D. Vascular calcification was observed in some of these studies, but not all (Zemplenyi and Mrhova, 1965; Kudejko, 1968; Taura et al., 1979; Kunitomo et al., 1981; Toda et al., 1983, 1985). Moreover, 1α,25-dihydroxyvitamin D_3_, the active form of vitamin D, stimulated vascular calcification by *in vitro* by reducing the expression of parathyroid hormone-related peptide as well as stimulating alkaline phosphatase activity in bovine vascular smooth muscle cells (Jono et al., 1998). On the other hand, there is a large body of research from clinical studies in humans indicating that low levels of serum 25-hydroxy vitamin D are associated with atherosclerosis (Reis et al., 2009; Carrelli et al., 2011; Shanker et al., 2011; Cheraghi et al., 2012). In line with this, the incidence of osteoporosis, a disease known to be related to vitamin D inadequacy, correlates with the incidence of atherosclerosis (Stojanovic et al., 2011). Therefore, different mechanisms may account for the promotion of atherogenesis by high and low vitamin D levels, respectively, and calcification may be crucial in the case of hypervitaminosis. Moreover, differences between the animal and human system may account for the conflicting results.

With respect to atherogenesis, 1α,25-dihydroxyvitamin D_3_ has been demonstrated to reduce macrophage adhesion and migration as well as foam cell formation in monocytes isolated from type 2 diabetic patients (Oh et al., 2012; Riek et al., 2013a,b). Mechanistic investigations in the context of these studies attributed the beneficial effects of vitamin D to a reduction of endoplasmatic reticulum stress in macrophages. This has been investigated in two mouse models, where vitamin D deficiency facilitated atherosclerosis, which could be reversed in the course of macrophage endoplasmatic reticulum stress suppression (Weng et al., 2013). Further evidence on beneficial effects of calcitriol treatment on atherosclerosis development has been obtained from an investigation with apolipoprotein E knock-out mice. In this study, oral calcitriol treatment decreased the production of proinflammatory chemokines, led to a reduced amount of inflammatory effector cells in atherosclerotic plaques and simultaneously increased amounts of regulatory T cells (Takeda et al., 2010). A similar link between vitamin D, T cell modulation, and atherosclerosis has also been established in humans with chronic kidney disease (CKD) (Yadav et al., 2012).

The renin-angiotensin-system is known for its detrimental effects on the cardiovascular system and has been shown to play an important role in the development of atherosclerosis. Interestingly, numerous studies in mice document that vitamin D signaling suppresses the renin-angiotensin-system and that vitamin D deficiency is associated with an increased activity of the renin-angiotensin-system (Li et al., 2002; Zhou et al., 2008; Szeto et al., 2012; Weng et al., 2013). Moreover, the inverse associations which are described for vitamin D and the occurrence of inflammatory cytokines, C-reactive protein, and adhesion molecules suggest a inhibitory role for vitamin D in the genesis of atherosclerosis (Brewer et al., 2011). Additionally, there is experimental evidence that vitamin D reduces the expression of matrix metalloproteinases that are involved in vascular calcification (Nakagawa et al., 2005; Qin et al., 2006).

However, there are also studies that found no evidence for an association between low vitamin D and atherosclerosis in patients suffering from different autoimmune diseases (Mok et al., 2012; Sachs et al., 2013). Similarly, there was no evidence for an association of *Bsm*I polymorphism, an intronic single nucleotide variation of the VDR gene, with atherosclerosis (El-Shehaby et al., 2013). Yet, it has been shown that atherosclerosis in monkeys is associated with low levels of VDR expression in coronary arteries even in the presence of higher plasma vitamin D concentrations (Schnatz et al., 2012a,b). Moreover, the activation of vitamin D can occur locally in macrophages that infiltrate atherosclerotic lesions and local vitamin D response might thus not necessarily correlate with serum vitamin D levels (Richart et al., 2007).

## 1α,25(OH)_2_D_3_ and autoimmune diseases

It is well established that vitamin D plays an important role in the regulation of immune functions (Schwalfenberg, 2011; Zhang et al., 2013a). Accordingly, several inflammatory autoimmune diseases like rheumatic disorders and type 1 diabetes have been associated with vitamin D deficiency (Adorini and Penna, 2008; Shapira et al., 2010). Inflammatory processes in the central nervous system are a hallmark of the autoimmune disease multiple sclerosis (MS) (Deckx et al., 2013). Several studies indicate that MS patients have lower levels of vitamin D and that higher vitamin D levels as well as vitamin D supplementation have a protective effect against MS (Munger et al., 2004, 2006; Ozgocmen et al., 2005). Moreover, vitamin D levels have been shown to vary in concordance with MS exacerbations (Correale et al., 2009) and it is possible that low vitamin D levels are rather a consequence of the sun avoidance of MS patients and not a direct cause of the disease (Munger et al., 2006). Regarding the effectiveness of vitamin D supplementation in the course of MS treatment, there are studies in mice and humans that suggest a beneficial effect of treatment (Goldberg et al., 1986; Wingerchuk et al., 2005; Pedersen et al., 2007; Burton et al., 2010). Interestingly, a gender specific effect of vitamin D has been demonstrated in mice and humans, which points to greater effects of vitamin D in females (Spach and Hayes, 2005; Correale et al., 2010).

Overall, there have been only a few controlled trials documenting the outcome of vitamin D supplementation on disease activity in rheumatic conditions, and the role of vitamin D in rheumatoid arthritis is therefore controversially discussed (Gatenby et al., 2013). Yet, a metaanalysis of observational studies on the vitamin D intake and vitamin D serum levels suggests an inverse association with rheumatoid arthritis (Song et al., 2012). Additionally, *in vitro* experiments with macrophages from healthy donors and rheumatoid arthritis patients indicate an enhanced anti-inflammatory potential of vitamin D in macrophages from the latter group (Neve et al., 2013).

It has been shown that the onset of autoimmunity in type 1 diabetes is preceded by a proinflammatory metabolic serum profile (Knip and Simell, 2012). Concurrently, a study in Italian children revealed reduced vitamin D serum levels in children at the onset of type 1 diabetes compared to children hospitalized for other reasons (Franchi et al., 2013). In conformity with these findings, metaanalyses suggest an association between vitamin D intake in early life and susceptibility for type 1 diabetes (Zipitis and Akobeng, 2008; Dong et al., 2013).

For inflammatory bowel disease (IBD), another autoimmune disorder, similar associations to that described above regarding vitamin D status and sunlight exposure have been reported (Garg et al., 2012; Ananthakrishnan, 2013). Animal studies in vitamin D deficient and VDR knockout (KO) mice reveal a dysregulation of T cells that might be of importance in the pathogenesis of IBD (Ooi et al., 2012).

In summary, there is considerable evidence for an association between vitamin D deficiency and inflammatory diseases. However, regarding the causality of this association and the benefit of vitamin D supplementation, only limited information is available and the existing data are still inconsistent.

## Interference of 1α,25(OH)_2_D_3_ with pro-inflammatory transcription factors and signaling pathways

Cell type specific up-regulation of proinflammatory genes and down-regulation of anti-inflammatory genes is a hallmark of the onset of an inflammatory reaction. Depending on the cell type, up-regulation of certain cytokines or enzymes which generate mediators of inflammation can occur at the transcriptional or posttranscriptional level. In addition, there is considerable crosstalk between various pathways which allows adaptation of the host defense reactions to the environment. According to their functions, the regulators of inflammatory reactions can be receptors such as toll like receptors, signal transducers as well as transcription factors which translate the activation of certain signal cascades into gene transcription. Additionally, regulation of gene expression during inflammatory processes can also occur on posttranslational level which is not focus of this review.

**Figure 1:**
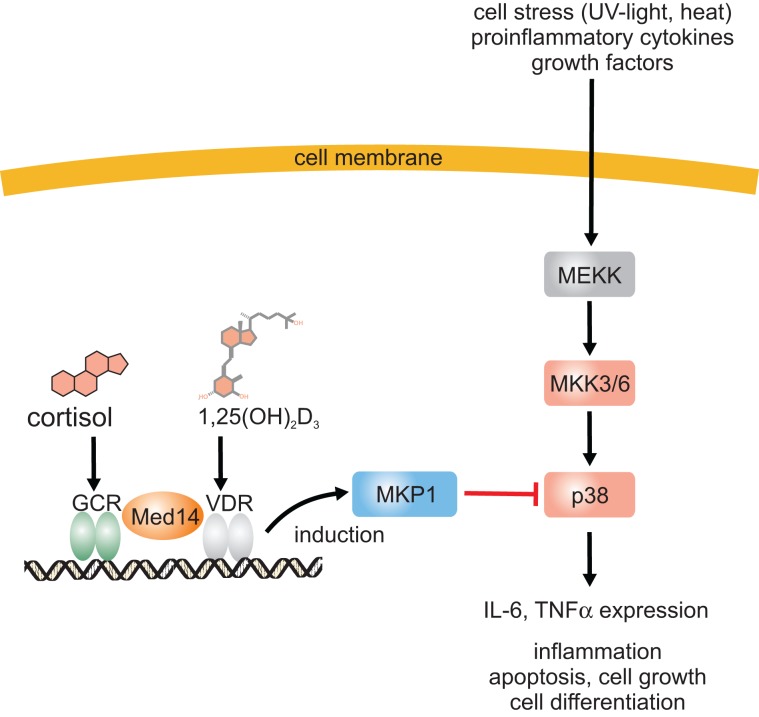
**Inhibition of the p38 MAP kinase pathway by 1α,25(OH)_2_D_3_ and a mechanism for the synergistic anti-inflammatory effects of 1α,25(OH)_2_D_3_ and glucocorticoids**. Proinflammatory stimuli lead to p38 MAP kinase phosphorylation and activation which subsequently induces expression of many proinflammatory proteins such as IL-6 and TNFα. 1α,25(OH)_2_D_3_ induces MKP1 expression which dephosphorylates and inactivates p38 MAP kinase. 1α,25(OH)_2_D_3_ stimulates glucocorticoid-induced MKP1 expression via enhanced expression of Med14.

**Figure 2:**
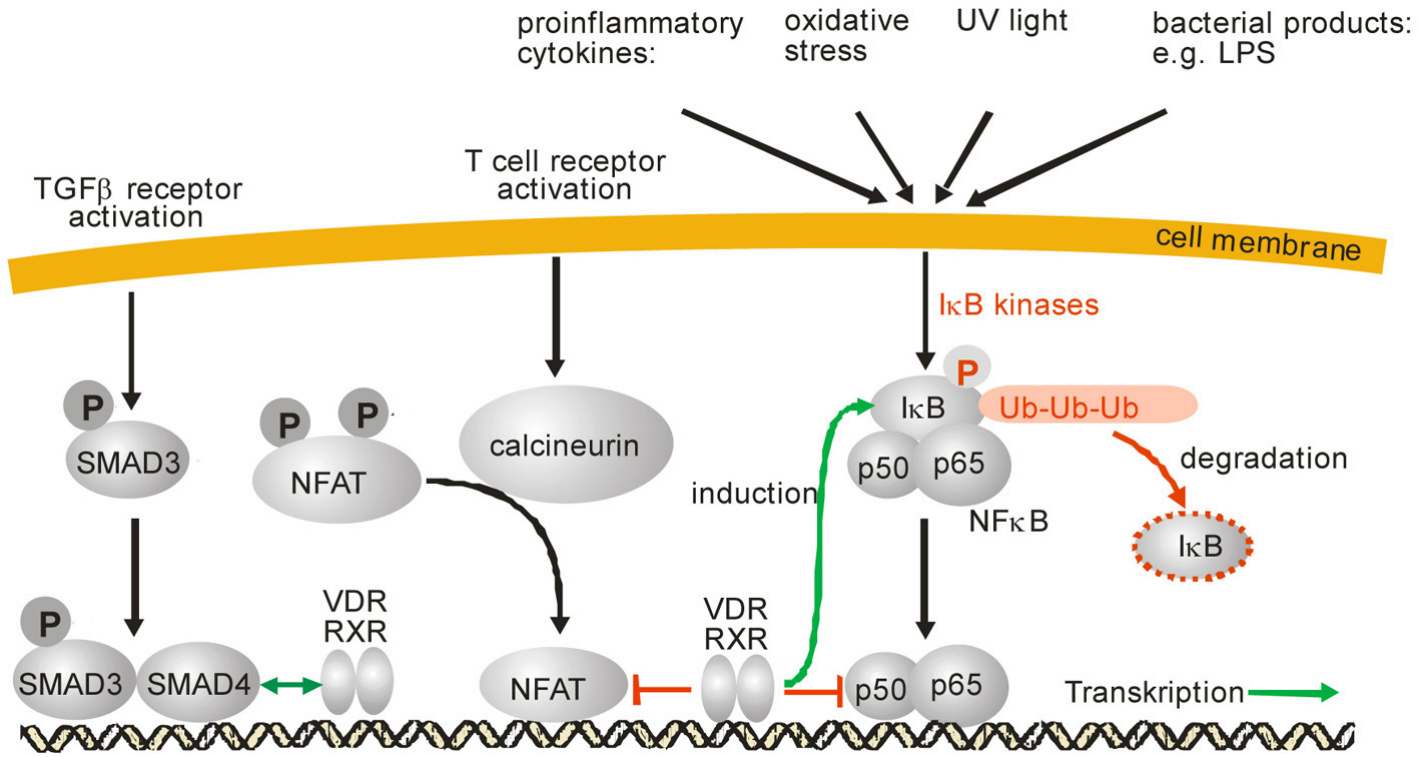
**SMAD, NFAT and NFκB signaling and modulation of these signaling pathways by 1α,25(OH)_2_D_3_, respective VDR/RXR**. IκB phosphorylation after various cell stress signals leads to its ubiquitinylation and subsequent proteosomal degradation. After IκB degradation, NFκB is released and translocates into the nucleus where it binds to DNA and modulates gene expression. Activation of NFAT is mediated by the protein phosphatase calcineurin which dephosphorylates NFAT. After dephosphorylation, NFAT translocates into the nucleus, interacts with a variety of other transcription factors and modulates gene expression. Activation of TGFβ receptors leads to phosphorylation of SMAD2 and SMAD3 as well as subsequent translocation into the nucleus. SMAD3 forms a complex with SMAD4 and modulates gene expression of its target genes. After activation by 1α,25(OH)_2_D_3_ the VDR/RXR heterodimer can inhibit NFκB signaling either by induction of IκB or by interference with NFκB DNA binding. Also, inhibition of NFAT signaling was reported by prevention of NFAT binding to its response elements.

At the level of intracellular signal transduction, MAP kinases such as JNK or p38 have been identified as central signal transducers of inflammatory signals. Interestingly, it has been observed that there is a cross talk between VDR/RXR and MAP kinase signaling on many levels and the outcome, e.g., stimulation or inhibition, depends on the stimulus, cell type and the response (Miodovnik et al., 2012). Regarding inflammation, it has become obvious that vitamin D inhibits production of proinflammatory cytokines like IL-6 or TNFα in monocytes via the inhibition of p38 MAP kinase (Zhang et al., 2012). Inhibition of p38 in monocytes was found to be due to induction of MAPK phosphatase-1 (MKP1) which dephosphorylates p38 and thus reduces p38 activation (Figure 1). A similar mechanism was found in prostate cells where induction of MKP5 by 1α,25(OH)_2_D_3_ was responsible for down-regulation of IL-6 mRNA expression (Nonn et al., 2006). 1α,25(OH)_2_D_3_ increases MKP5 transcription by induction of VDR/RXR binding to a VDRE in the MKP5 promoter. Beside this indirect modulation of signaling cascades, 1α,25(OH)_2_D_3_ and its receptor complex VDR/RXR can interact with other transcription factors such as NF-κB, nuclear factor of activated T-cells (NFAT), or the glucocorticoid receptor (GCR) which leads to anti-inflammatory effects (Figure 2). Activation of VDR inhibits NF-κB activation and signaling. NFκB is a ubiquitously expressed transcription factor which represents a heterodimer. In the inactive state it interacts with IκB which keeps it in the cytosol (Karin and Lin, 2002). Upon cell activation by proinflammatory stimuli, IκB is phosphorylated and subsequently ubiquitinylated, which leads to proteasomal degradation of the IκB protein. Free NFκB translocates to the nucleus where it activates transcription of proinflammatory cytokines, antiapoptotic factors as well as of enzymes involved in the generation of proinflammatory mediators such as COX-2 (Karin and Lin, 2002; Tsatsanis et al., 2006). It has been shown that 1α,25(OH)_2_D_3_ down-regulates NF-κB levels in lymphocytes (Yu et al., 1995) and that the vitamin D analog TX 527 prevents NF-κB activation in monocytes (Stio et al., 2007). Inhibition of NFκB activation by 1α,25(OH)_2_D_3_-mediated up-regulation of IκB expression was reported in human peritoneal macrophages (Cohen-Lahav et al., 2006) (Figure 2). Additionally, interference of vitamin D signaling with DNA binding of NFκB was found (Harant et al., 1998). It was shown that 1α,25(OH)_2_D_3_ inhibits NF-κB activity in human MRC-5 fibroblasts but not translocation of its subunits p50 and p65. The partial inhibition of NFκB DNA binding by 1α,25(OH)_2_D_3_ was dependent on de novo protein synthesis, suggesting that 1α,25(OH)_2_D_3_ may regulate expression of cellular factors which contribute to reduced DNA binding of NFκB (Harant et al., 1998). Thus, it seems that vitamin D is able to inhibit NFκB activation as well DNA binding (Figure 2).

Another interesting target for the anti-inflammatory signaling of vitamin D is NFAT (Figure 2). This transcription factor is activated by dephosphorylation by calcineurin which leads to translocation of this protein and transcriptional activation of proinflammatory genes such as interleukin 2 and cyclooxygenase-2 (Duque et al., 2005; Muller and Rao, 2010). In T-lymphocytes, it was shown for the interleukin 2 promoter that VDR-RXR heterodimers bind to an NFAT binding site and thus inhibit NFAT activity (Takeuchi et al., 1998). Similar data were obtained for interleukin 17 where 1α,25(OH)_2_D_3_ blocked NFAT activity which contributed to repression of interleukin17A expression in inflammatory CD4^+^ T cells by the hormone (Joshi et al., 2011).

Another interesting finding was that vitamin D enhances the anti-inflammatory activities of GCs (Figure 1). The strong anti-inflammatory activities of GCs are mediated by the GCR. It belongs to the nuclear receptor family. Upon ligand binding the receptor dimerizes and translocates into the nucleus where it binds to GC-responsive elements (GRE) and modulates gene expression (Barnes, 1998). In general, GCs down-regulate expression of pro-inflammatory genes and up-regulate anti-inflammatory genes. It was found in asthmatics that dexamethasone-induced MKP-1 expression as a marker for GC responsiveness is significantly increased when serum vitamin D levels increase suggesting that vitamin D may enhance GC responsiveness (Sutherland et al., 2010). It is interesting to note that MKP-1 is also a vitamin D target gene as mentioned above (Zhang et al., 2012). Vitamin D enhancement of GC-induction of MKP1 was abolished both in purified CD14^+^ and CD14^−^ cells and it was found that the synergism depends on vitamin D-induced GM-CSF release from CD14^−^ cells and GM-CSF-dependent MED14 induction in CD14^+^ cells (Zhang et al., 2013b). MED14 is part of the mediator complex involved in the regulation of transcriptional initiation and it was found to form a complex with VDR and mediate ligand-dependent enhancement of transcription by the VDR (Rachez et al., 1999) (Figure 1). Interestingly, MED14 also enhances gene activation by the GCR in a gene-specific manner (Chen et al., 2006). For MKP1 it was found in human monocytes that VDR and GCR bind to a corresponding VDRE and two GREs after ligand stimulation (Figure 1). After GM-CSF treatment, MED14 was recruited to the promoter after addition of 1α,25(OH)_2_D_3_ but not dexamethasone indicating that MED14 recruitment depends on the VDR (Zhang et al., 2013b). 1α,25(OH)_2_D_3_ enhanced the binding of the GCR to the GRE in close proximity to the VDRE in the presence of GM-CSF and ChIP analysis suggest a MED14-VDR-GCR complex at the MKP1 promoter with bridges the crosstalk between vitamin D and GCs (Zhang et al., 2013b). The data from single gene analyses such as MKP1 suggest that the VDR interacts with other signaling pathways.

At present there are genome-wide data available from immortalized lymphoblastoid cell lines (Ramagopalan et al., 2010), undifferentiated and LPS stimulated THP-1 cells (Heikkinen et al., 2011; Tuoresmäki et al., 2014), LS180 colorectal cancer cells (Meyer et al., 2012) and LX2 hepatic stellate cells (Ding et al., 2013). These six ChIP-seq data sets showed 21,776 non-overlapping VDR binding sites whereas only 54 sites were common in all six data sets. The data suggest that, apart from a few sites, VDR binding is strongly cell and stimulus specific. In the non-overlapping binding sites, only 17.5% contain a DR3-type VDRE whereas the percentage of DR3-type response elements is enriched in highly ligand-responsive loci. All these data suggest that the VDR interacts with other transcription factors and that these interactions might only be in part ligand dependent. Regarding inflammation, the genome-wide effects of LPS on VDR location in THP-1 cells are of special interest (Tuoresmäki et al., 2014). From the 805 VDR binding sites, only 462 overlap in untreated and LPS-treated THP cells which were stimulated with 1α,25(OH)_2_D_3_. Thus, LPS treatment leads to a considerable change in VDR location. In THP-1 cells, bioinformatic searches for shared binding sites revealed motifs for CEBP1, PU.1 in stimulated THP-1 cells whereas NFYA, LHX3-like and NANOG were found for unstimulated cells but no transcription factor has been identified in conjunction with LPS treatment. Of note, binding sites for JUN, a component of the AP1 transcription factor, were found to be enriched at VDR loci in LX2 hepatic stellate cells. This is of interest regarding inflammation as AP1 is known to be a transcription factor that regulates expression of many proinflammatory genes. At present, there are many data available on single gene levels but there is still a missing link between these data and the genome-wide observations. Since VDR signaling seems to be strongly cell type and stimulus-dependent, more genome-wide data with different cell types and stimuli are required to understand the mechanisms how 1α,25(OH)_2_D_3_ modulates gene expression under inflammatory conditions.

## Regulation of the expression of proinflammatory enzymes by 1α,25(OH)_2_D_3_

Arachidonic acid derived eicosanoids which comprise prostaglandins and leukotrienes play an important role in inflammatory processes (Harizi et al., 2008). Of the enzymes involved in prostaglandin synthesis, cyclooxygenase-2 (COX-2) and microsomal prostaglandin E synthase 1 (mPGES-1) have been shown to be induced in many inflammatory conditions (Tomasoni et al., 1998; Murakami et al., 2000; Cipollone and Fazia, 2006; Petrovic et al., 2006) and inhibition of both enzymes is a common approach in the treatment of inflammatory diseases (Fahmi, 2004; Ramalho et al., 2009; Dallaporta et al., 2010).

In prostate cancer cells it has been demonstrated that 1α,25(OH)_2_D_3_ inhibits the expression of COX-2 on mRNA and protein level as well as the expression of prostaglandin receptors on mRNA level and simultaneously upregulates prostaglandin catabolism via 15-hydroxyprostaglandin dehydrogenase (Moreno et al., 2005). In addition, the combination of calcitriol with COX-inhibitors led to synergistic growth inhibition (Moreno et al., 2005). Similar results were obtained with the combination of 1α,25(OH)_2_D_3_ and COX-inhibitors in different leukemia cells (Jamshidi et al., 2008). In accordance with the previous findings, treatment with the vitamin D analog elocalcitol resulted in decreased COX-2 expression and diminished PGE_2_ synthesis in prostate cells (Penna et al., 2009). The COX-2/PGE_2_-pathway was also identified as the mediator of the growth inhibitory effect of calcitriol in breast cancer cells (Yuan et al., 2012). Furthermore, COX-2 upregulation in placental trophoblasts in response to oxidative stress and in myometrial cells in response to interleukin-1β was inhibited by 1α,25(OH)_2_D_3_ (Sun et al., 2013; Thota et al., 2013).

Thill et al. found correlations between VDR expression and expression of COX-2 as well as 15-hydroxy PG dehydrogenase in malignant breast cells and in cells from female reproductive tissues (Thill et al., 2009, 2010, 2012).

In human lung fibroblasts inhibition of PGE_2_-production by vitamin D was found which was not due to altered COX-expression. Yet, vitamin D inhibited IL-1β-induced mPGES-1 expression and simultaneously stimulated 15-hydroxy PG dehydrogenase (Liu et al., 2014).

5-lipoxygenase (5-LO) accounts for the first two steps in leukotriene biosynthesis. Leukotrienes exert potent proinflammatory actions and have been associated with several chronic inflammatory diseases (Haeggstrom and Funk, 2011).

In the myeloid cell line HL-60, treatment with 1α,25(OH)_2_D_3_ triggers differentiation into monocytic cells. Simultaneously, 1α,25(OH)_2_D_3_ has been shown to induce 5-LO expression on mRNA and protein level as well as to increase 5-LO enzyme activity (Bennett et al., 1993; Brungs et al., 1994). A similar effect was also observed in the monocytic cell line Mono Mac 6. Additionally, this effect was strongly enhanced by the combination of 1α,25(OH)_2_D_3_ with transforming growth factor β (TGF-β) (Brungs et al., 1995; Harle et al., 1998). Mechanistically, the effect of 1α,25(OH)_2_D_3_ on 5-LO expression was related to VDR binding sites in the 5-LO promoter and distal parts of the 5-LO gene (Sorg et al., 2006; Stoffers et al., 2010) and is due to stimulation of 5-LO transcript elongation (Stoffers et al., 2010).

Previous results suggest a modulatory role of vitamin D in the inflammatory response of cells of the monocyte/macrophage lineage, which is again modulated by TGF-β. In this context, it is interesting that macrophages contain 1α-hydroxylase and therefore are capable of autocrine or paracrine activation of vitamin D (Lagishetty et al., 2011). Moreover, in keratinocytes autocrine TGF-β production is induced by vitamin D (Kim et al., 1992). Crucial participation of monocytes/macrophages in diverse inflammatory processes has been demonstrated (Cutolo, 1999; Yoon and Jun, 1999; Moore et al., 2013). Besides induction of 5-lipoxygenase, the combination of TGF-β and 1α,25(OH)_2_D_3_ has been shown to induce the differentiation antigen CD69 in monocytic cells (Wobke et al., 2013). Overexpression of CD69 again, has been shown in the context of local dermal inflammation, systemic lupus erythematosus, hyperthyroid Graves' disease and autoimmune thyroiditis (Fernandez-Herrera et al., 1996; Portales-Perez et al., 1997; Crispin et al., 1998; Gessl and Waldhausl, 1998).

## 1α,25(OH)_2_D_3_ as regulator of cytokine gene expression, protein production/release and signaling

### TGF-β and Smad signaling in inflammation and the influence of 1α, 25(OH)_2_D_3_

TGF-β is a pleiotropic cytokine with a broad range of biologic effects, which is involved in the regulation of inflammatory processes on several levels. A main mechanism in this respect is the maintenance of T cell tolerance to self or innocuous antigens (Li and Flavell, 2008). In cancer-associated inflammation, TGF-β suppresses the anti-tumor activity of diverse immune cells, including T-cells, natural killer (NK) cells, neutrophils, monocytes and macrophages (Bierie and Moses, 2010). A great number of studies focused on the role of TGF-β in fibrosis and associated inflammation. In these diseases, TGF-β regulates influx and activation of immune cells, as well as the actual fibrotic process, and thus the delicate balance between an appropriate inflammatory response and the development of pathologic fibrosis (Flanders, 2004; Sheppard, 2006; Lan, 2011). Several mechanistic links between inflammation and fibrosis are known, but the complete picture remains to be established (Lee and Kalluri, 2010). TGF-β signaling in these processes has been attributed both to canonical TGF-β signaling via the Smad proteins (signal-dependent transcription factors) as well as non-Smad signaling pathways (e.g., via MAPK pathways) (Figure 2).

Independent of inflammatory model systems, 1α,25(OH)_2_D_3_ and TGF-β/Smad signaling pathways have been found to be interrelated through three mechanisms: (i) the existence of a common regulator protein, the oncoprotein Ski, which can repress both pathways (Ueki and Hayman, 2003), (ii) the possibility of joint gene regulation via VDR and Smad recognition elements that are located in close proximity to a target promoter (Subramaniam et al., 2001) (Figure 2) or (ii) direct interaction of Smad3 and vitamin D signaling, whereby Smad acts as a coregulator specific for ligand-induced VDR transactivation (Yanagisawa et al., 1999).

The influence of vitamin D on inflammation-related signaling via TGF-β and Smad has mainly been investigated in models of fibrosis, and distinct mechanisms have been elucidated. Activation of 1α,25(OH)_2_D_3_ signaling by the natural ligand itself or its synthetic analogs reduces TGF-β expression (Kim et al., 2013) and interferes with the downstream signaling. The latter occurs via several mechanisms: downregulation of phosphorylated activatory Smads (Smad2/3 and 4) accompanied by upregulation of inhibitory Smad6 (Kim et al., 2013) (Figure 2); an inhibitory interaction between 1α,25(OH)_2_D_3_-bound VDR and Smad3 (Ito et al., 2013) or inhibition of Smad2 phosphorylation and nuclear translocation of Smad2/3, coincident with inhibited protein expression from TGF-β target genes (Halder et al., 2011). Similar findings have been made in studies with nephropathy models where suppression of TGF-β and p-Smad2/3 expression (Xiao et al., 2009) or a decrease in Smad2 and an increase in inhibitory Smad7 (Hullett et al., 2005) have been detected. In a large-scale study using hepatic stellate cells, TGF-β has been shown to cause chromatin remodeling events that led to a redistribution of genome-wide VDR binding sites (the VDR cistrome) with a shift toward VDR binding to Smad3-dependent, profibrotic target genes. In this study, VDR ligands led to a reduced Smad3 occupancy at these genes and thus antifibrotic effects (Ding et al., 2013). Although hepatic stellate cells do not belong to the immune system, and the interplay between VDR and TGF-β/Smad signaling may be dependent on the cell type, key aspects of this elaborate study deserve mention. More than 10^4^ genomic sites were found to be co-occupied by both VDR and SMAD3 in these cells, and an analysis of the spatial relationships between the two transcription factors revealed that the respective response elements were located within a range of 200 base pairs (one nucleosomal window). Mechanistically, TGF-β signaling seems to deplete nucleosomes from the co-occupied sites and thus allow access of VDR to these sites. Vitamin D signaling on the other hand seems to limit TGF-β activation by inhibited coactivator recruitment. Spatiotemporal analysis revealed that 1α,25(OH)_2_D_3_/TGF-β-induced VDR and SMAD3 binding to the co-occupied sites were inversely correlated. The maximum of SMAD3 binding occurred 1 h after treatment and was reduced by 70% after 4 h, when VDR binding was maximal. Therefore, TGF-β signaling seems to change the chromatin architecture in a way in which liganded VDR can reverse Smad activation.

### The influence of 1α,25(OH)_2_D_3_ on interleukin (IL) gene expression and signaling

The finding that 1α,25(OH)_2_D_3_ interacts with the production of interleukins (Tsoukas et al., 1984) is of certain interest in the history of vitamin D research, as a crucial finding that expanded the view to roles beyond calcium homeostasis and crucially contributed to establish an immunoregulatory function of vitamin D (Tsoukas et al., 1984).

The interleukins are a large group of cytokines of central importance for the intercellular communication between the different cells generally involved in inflammatory responses. These cells mainly encompass the leukocytes in their various stages of differentiation (distinct T-cells subsets, monocytes, macrophages, dendritic cells (DCs), granulocytes and B-lymphocytes) and cells of the connective tissue and vasculature (fibroblasts, endothelial cells). Furthermore, in specific organ-related diseases with inflammatory components (psoriasis, CKD, placental infection/inflammation, obesity, and others), further cell types are involved, e.g., keratinocytes, endothelial cells, trophoblasts, and adipocytes. All of them are capable of synthesizing interleukins, and the influence of 1α,25(OH)_2_D_3_ on IL gene expression has been investigated. The influence of 1α,25(OH)_2_D_3_ on IL gene expression and signaling in the different cell types will be outlined in the following.

#### Leukocytes

Several studies, especially the early ones, included *ex vivo* experiments with cellular samples from healthy individuals, mainly with PBMC (Rigby et al., 1984; Tsoukas et al., 1984; Saggese et al., 1989; Muller and Bendtzen, 1992), (partly) isolated T-cells (Bhalla et al., 1986), (partly) isolated monocytes (Bhalla et al., 1986; Muller et al., 1992; Zarrabeitia et al., 1992; Lemire et al., 1995; Lyakh et al., 2005), or cocultures of T-cells and monocytes (Tsoukas et al., 1989).

#### PBMC and T-cells

In stimulated PBMC, as a preparation that includes different cell types, 1α,25(OH)_2_D_3_ caused suppression of IL-2 production (Rigby et al., 1984; Tsoukas et al., 1984; Saggese et al., 1989) and reduced release of IL-1β, IL-6, and IL-10 (Joshi et al., 2011). Furthermore, the vitamin D analog paracalcitol led to reduced IL-8 production in stimulated PBMC (Eleftheriadis et al., 2010).

**Figure 3:**
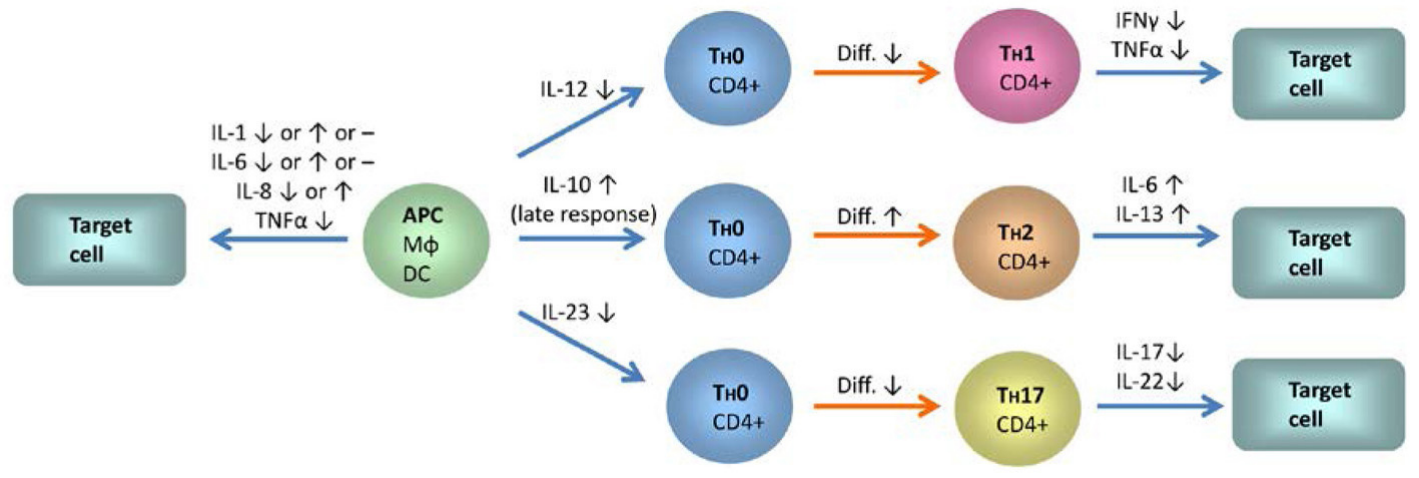
**The influence of 1α,25(OH)_2_D_3_ on the expression of interleukins, TNFα and IFNγ in monocytes, dendritic cells, and different T-cell subsets**. Blue arrows indicate IL signaling between the different cell types and red arrows indicate differentiation processes. IL-12 and IL-23 expression is downregulated in monocytes and dendritic cells by 1α,25(OH)_2_D_3_. In contrast, IL-10 expression is enhanced. A shift from a Th1 profile toward the Th2 type and a decrease in Th17 responses is to be anticipated from these changes. Inhibition of T-cell autoregulation by 1α,25(OH)_2_D_3_-mediated suppression of IL-2 expression is not shown. Abbreviations and symbols: APC, antigen presenting cell; MΦ, macrophage; DC, dendritic cell; ↑, upregulation; ↓, downregulation; -, no changes.

In more cell specific experiments with (partly) isolated T-cells, 1α,25(OH)_2_D_3_-mediated inhibition of IL-2 mRNA synthesis induced by lectin/phorbol ester (Matsui et al., 1986) or protein production induced by lectin (Bhalla et al., 1986), was confirmed. This was also observed for the two subsets of CD4^+^ and CD8^+^ T-cells (Jordan et al., 1989), which however displayed stimulus-dependency for IL-2 protein production. In a more detailed analysis, IL production by CD4^+^ and CD8^+^ cells was studied by flow cytometry on single cell level. In both populations, a decrease in IL-2 production was found. Conversely, regarding other IL class members analyzed in the same study, 1α,25(OH)_2_D_3_ increased the low percentage of IL-13-producing cells in both subsets and IL-6 producing CD4^+^ and CD8^+^ T-cells could only be detected after incubation with 1α,25(OH)_2_D_3_ (Willheim et al., 1999) (Figure 3).

The finding that IL-2 gene expression is reduced by 1α,25(OH)_2_D_3_ in T-cells has moreover been confirmed in two studies using the human T-cell line Jurkat, and the mechanisms have been studied. It has been found that the VDR seems to cause direct transcriptional repression of IL-2 gene expression via blockage of a positive regulatory element recognized by the transcription factor NFAT within the IL-2 promoter (Alroy et al., 1995). In a later study, the repression has been kinetically classified as a primary response to 1α,25(OH)_2_D_3_, and ligand-dependent VDR binding at the IL-2 gene locus was detected using ChIP assays (Matilainen et al., 2010b) (Figure 2). It has to be mentioned, however, that long term pretreatment of Jurkat cells with 1α,25(OH)_2_D_3_ before stimulation with mitogen and phorbol ester seems to enhance IL-2 mRNA expression (Prehn and Jordan, 1989). Studies using T-cells from other species confirmed the inhibitory effect of 1α,25(OH)_2_D_3_ on IL-2 production (Hodler et al., 1985).

Similar findings as for IL-2 have been made regarding the inhibition of IL-17 production by 1α,25(OH)_2_D_3_ from T-cells in a more recent report. It has been found that (i) the VDR competes for binding with NFAT and recruits histone deacetylase (HDAC) to the human IL-17 promoter, thus inhibiting its activation, (ii) binding of the activatory transcription factor Runx1 to the mouse IL-17A promoter was inhibited through sequestration of Runx1 by the VDR in the presence of 1α,25(OH)_2_D_3_ and (iii) 1α,25(OH)_2_D_3_ induced the IL-17 inhibiting transcription factor Foxp3 (Joshi et al., 2011). Other studies suggest a post-transcriptional mechanism of IL-17 inhibition by VDR via induction of the translation inhibitor C/EBP homologous protein (CHOP) (Chang et al., 2010).

Apart from studies with PBMC or T-cells from healthy individuals or experiments with cell lines, a few studies exist with cell samples from patients suffering from inflammatory diseases. In contrast to the findings with cells from healthy individuals after 1α,25(OH)_2_D_3_ treatment, PBMC isolated from hemodialysis patients responded to treatment with 1α(OH)D_3_ by enhanced IL-2 protein production, however, starting from a significantly lower level of IL-2 production compared to healthy controls (Tabata et al., 1988). The capacity of PBMC from Crohn's disease patients to produce IL-6 has been elevated by 1α,25(OH)_2_D_3_ treatment of the patients (Bendix-Struve et al., 2010). IL-6 and IL-8 production and mRNA expression have been found to be decreased by 1α,25(OH)_2_D_3_ in stimulated PBMC of psoriatic patients (Inoue et al., 1998). In PBMC from treatment-naive patients with early rheumatoid arthritis (RA), reduced IL-17A and increased IL-4 levels have been observed in the presence of 1α,25(OH)_2_D_3_. In the FACS-separated subpopulation of memory T-cells (CD45RO+), 1α,25(OH)_2_D_3_ suppressed IL-17A, IL-17F and IL-22 (Colin et al., 2010) (Figure 3).

#### Monocytes

In an early report, IL-1 production by human monocytes/macrophages enriched from PBMC has been found to be elevated by single 1α,25(OH)_2_D_3_ treatment (Bhalla et al., 1986). In subsequent studies with stimulated, monocyte-enriched cultures from PBMC, either no 1α,25(OH)_2_D_3_ effect has been detected (Zarrabeitia et al., 1992) or a reduction of IL-1 (and IL-6) production has been found, which seemed to be based on post-transcriptional events (Muller et al., 1992). The decrease in IL-1 production has been confirmed for co-cultures of T-cells and monocyte-enriched PBMC (Tsoukas et al., 1989). However, it has to be pointed out that different stimuli to elicit IL-1 production had been used in these studies. In human monocytic cell lines, (U937, HL-60 or THP-1), no induction (THP-1), or upregulation of IL-1β mRNA (U937, HL-60) by 1α,25(OH)_2_D_3_ has been detected, which varied with the presence or absence and the type of the co-stimulus that was used (phorbol ester, lipopolysaccharide) (Bhalla et al., 1991; Blifeld et al., 1991; Fagan et al., 1991). Further it is noteworthy that conflicting data exist for studies with U937 cells regarding the actual secretion of IL-1β protein (Blifeld et al., 1991; Fagan et al., 1991; Taimi et al., 1993). In THP-1 cells stimulated with agonists for Toll-like receptor 8, IL-1β mRNA was induced and could be suppressed by 1α,25(OH)_2_D_3_ (Li et al., 2013).

In a more recent study, expression of IL-1 and IL-6 mRNA in freshly isolated monocytes and macrophages cultured for 7 days has been investigated. Interestingly, IL-1 and IL-6 gene expression has been regulated differently in these two distinct stages of monocyte/macrophage maturation. In the monocytes, basal IL-1 and IL-6 mRNA expression has been found to be slightly upregulated by 1α,25(OH)_2_D_3_ treatment compared to untreated controls. For 1α,25(OH)_2_D_3_ treated monocytes that were additionally stimulated with LPS or TNFα, no or only marginal differences have been found compared to LPS or TNFα treatment without 1α,25(OH)_2_D_3_ preincubation. In contrast, 1α,25(OH)_2_D_3_ treatment reduced basal IL-1 and IL-6 levels in macrophages. In 1α,25(OH)_2_D_3_ treated macrophages that were additionally stimulated with LPS or TNFα, only TNFα-stimulated IL-6 mRNA expression was influenced, whereas no significant changes were observed for IL-1 and IL-6 after 1α,25(OH)_2_D_3_/LPS-treatment. These findings show that in monocytes/macrophages, the influence of 1α,25(OH)_2_D_3_ on IL expression depends on the type of IL under consideration, the degree of maturation, and the stimulus that is employed (Di Rosa et al., 2012). In a second recent investigation, significant inhibition of IL-6 mRNA expression and protein secretion was observed in PBMC, and subsequent FACS-based analysis revealed a concomitant decrease in CD14+ IL-6-producing monocytes (Zhang et al., 2012) (Figure 3).

Apart from the two prominent monokines IL-1 and IL-6, the synthesis of IL-3 has been found to be influenced by 1α,25(OH)_2_D_3_ in the murine monocytic cell line WEHI-3. However, whereas one report describes dose-dependent inhibition of IL-3 production in this cell line (Abe et al., 1986), the second finds concentration-dependent stimulation or inhibition of IL-3 production (Hodler et al., 1985). Furthermore, the interleukin family members IL-8, IL10, and IL-12 have been studied more intensely on mechanistic level.

IL-10 and IL-12-production by stimulated primary human monocytes has been found to be negatively regulated by 1α,25(OH)_2_D_3_ (Lemire et al., 1995; Lyakh et al., 2005). These two genes have been identified as primary 1α,25(OH)_2_D_3_ target genes as judged by rapid VDR recruitment detected via ChIP assays in the monocytic cell line THP-1 (Matilainen et al., 2010b). Further studies with this cell line include extensive mechanistic analyses regarding the influence of 1α,25(OH)_2_D_3_ on the expression of IL-8, IL-10, and IL-12B. The IL-8 gene has been shown to be an up-regulated, primary target gene, located within an insulated cluster of CXC motif ligand (CXCL) genes. IL-8 and its neighboring genes CXCL1 and CXCL6 seem to be under the control of a consensus VDR binding motif located 22 kb downstream of the IL-8 transcription start site, which mediates 1α,25(OH)_2_D_3_–dependent chromatin opening (Ryynanen and Carlberg, 2013). As discussed in this report, this finding is seemingly in contradiction with other studies (e.g., Di Rosa et al., 2012). These studies used different cells and foremost, cells were stimulated with agents like LPS that activate transcription factors, e.g., NF-κB, that are themselves regulated by 1α,25(OH)_2_D_3_. As described above, NF-κB activity is inhibited by 1α,25(OH)_2_D_3_ (Harant et al., 1998) (Figure 2). It has been put forward that 1α,25(OH)_2_D_3_ may have a dual effect: primary up-regulation of genes like IL-8, which supports the inflammatory response in the early phase of inflammation, e.g., by IL-8 production, and secondary effects which would help to shut down the inflammatory process, e.g., by inhibition of NF-κB-mediated pro-inflammatory responses (Ryynanen and Carlberg, 2013). This could explain that in another study in which THP-1 cells were used, no significant effect of 1α,25(OH)_2_D_3_ on IL-8 expression was found on protein level. In this study, the cells have been stimulated with LPS after only 2 h of 1α,25(OH)_2_D_3_ treatment before IL-8 protein was analyzed after 24 and 48 h (Kuo et al., 2010). Similarly, U937 cells exposed to high glucose (a condition which leads to different stress responses like NF-κB or MAPK activation) (Stan et al., 2011; Yang et al., 2013) showed lower IL-8 secretion after pretreatment with 1α,25(OH)_2_D_3_ (Jain and Micinski, 2013). Therefore, the interference of 1α,25(OH)_2_D_3_ with cell signaling pathways of inflammatory or cell stress responses, like NF-κB or MAPK activation, and differences in treatment schedules may explain the different findings. In contrast to IL-8 as an up-regulated gene, the primary effect of 1α,25(OH)_2_D_3_ on IL-10 expression is down-regulation, followed by up-regulation at a later stage (Figure 3). Cyclic binding of VDR to a distal promoter region with conserved VDREs, that loops 1α,25(OH)_2_D_3_-dependently to the transcription start site and induces epigenetic changes and chromatin remodeling, was detected (Matilainen et al., 2010a,b). IL-12B has been identified as a 1α,25(OH)_2_D_3_-dependently down-regulated gene in LPS-treated THP-1 cells. The gene harbors two VDR binding sites within ~6 kb upstream of the transcription start site to which the VDR and its partner retinoid receptor (RXR) recruit co-repressors and consequently induce epigenetic changes associated with gene repression (Matilainen et al., 2010b; Gynther et al., 2011). An earlier report attributed the down-regulation of IL-12 via interference of 1α,25(OH)_2_D_3_/VDR with NF-κB binding to proximal IL-12 promoter regions (D'Ambrosio et al., 1998). It has been suggested in the more recent report that this suppression of proximal sites is due to epigenetic changes at that location via the distal VDRE binding sites identified in the more recent study (Gynther et al., 2011) (Figure 3).

In addition to data from experiments with monocytes, macrophages, and DCs as differentiated members of the monocytic lineage have been investigated.

In macrophages from vitamin D-deficient mice, IL-1, and IL-6 production (evaluated as biological activity) was significantly reduced relative to control mice. Notably, this was paralleled by a decrease in macrophage cytotoxicity. Furthermore, the vitamin D deficient mice had reduced serum levels of IL-1 and IL-6 after challenge with LPS (Kankova et al., 1991). In human monocyte-derived macrophages and PMA-differentiated U937 cells, which were stimulated with LPS or PMA, IL-1β production was strongly stimulated by 1α,25(OH)_2_D_3_. This effect was ascribed to increased IL-1β transcription, but not by RNA stabilization, and seemed to be mediated by Erk1/2. Moreover, 1α,25(OH)_2_D_3_ induced the expression and phosphorylation of CCAAT enhancer-binding protein β as a known IL-1 β-regulating transcription factor (Lee et al., 2011). The upregulation of IL-1β by 1α,25(OH)_2_D_3_ is also relevant for infection-induced inflammation, as in THP-1 cells or primary human macrophages infected with *Mycobacterium tuberculosis* (as well as in non-infected controls), 1α,25(OH)_2_D_3_ increased the expression of IL-1β mRNA. IL-1β is a critical factor for host defense in this disease. Notably, mature intracellular IL-1β protein was only detected in infected, 1α,25(OH)_2_D_3_ treated THP-1 cells, which represents a further level of gene expression control exerted by 1α,25(OH)_2_D_3_. Secretion of IL-1β was only seen in infected cells, and significantly enhanced by 1α,25(OH)_2_D_3_. With respect to the mechanism, the study revealed 1α,25(OH)_2_D_3_–dependent binding of VDR to a promoter-proximal consensus VDRE, which was paralleled by upregulated VDR-expression, and recruitment of RNA polymerase II to the transcription start site (Verway et al., 2013).

In a further study with mouse macrophages, 1α,25(OH)_2_D_3_ led to reduced mRNA expression of the IL-12 subunit p40 in response to LPS/interferon gamma (IFNγ) stimulation (Korf et al., 2012), which is in line with the effects seen in monocytes, as described above (Figure 3). Stimulation of the macrophages with 1α,25(OH)_2_D_3_ was accompanied by upregulation of VDR and the 1α,25(OH)_2_D_3_–catabolic enzyme CYP24. Further changes concerned the potential to stimulate T-cells, as assessed by co-culture experiments including FACS analysis of surface markers. These effects could not be observed with IL-10 deficient macrophages. Notably, the effects on IL-12 p40 expression and T-cell stimulation also occurred in monocytes/macrophages from non-obese diabetic (NOD) mice, which have a background of inflammatory features seen in type 1 diabetes (Korf et al., 2012).

Analogous studies have been conducted for DCs from NOD mice or non- obese diabetes-resistant (NOR) control mice. In both cases, 1α,25(OH)_2_D_3_ altered the phenotype of DCs and inhibited the LPS/IFNγ–induced mRNA expression and protein secretion of IL-10 and IL-12 (Van Etten et al., 2004). In general, it has been shown that 1α,25(OH)_2_D_3_ prevents *in vitro* differentiation of human monocytes into immature DCs, associated with decreased capacity to activate T-cells. Furthermore, 1α,25(OH)_2_D_3_ inhibits maturation of DCs. In maturating DCs, 1α,25(OH)_2_D_3_ reduces IL-12p70 and enhances IL-10 secretion upon stimulation of the DCs by CD40-crosslinking (Penna and Adorini, 2000). This has been independently confirmed for IL-12p70 production upon LPS stimulation (Sochorova et al., 2009). Additionally, these findings are in line with a study on the generation of regulatory DCs for therapeutic use from human monocytes, which were differentiated in the presence of 1α,25(OH)_2_D_3_. Apart from reduced LPS-induced IL-12 and enhanced IL-10 secretion of the maturating cells, a major characteristic of these 1α,25(OH)_2_D_3_–treated DCs is their low level of IL-23 secretion, which was apparent with or without stimulation with LPS (Pedersen et al., 2009) (Figure 3). A further recent investigation used monocyte-derived DCs from Crohn's disease patients. When the cells were cultured in the presence of 25(OH)D_3_ or 1α,25(OH)_2_D_3_ and matured with LPS, they exhibited significantly increased IL-6 production, and non-significant reductions in and IL-10 and IL-12p70. IL-1β and Il-8 levels were not affected in this study (Bartels et al., 2013).

#### B-cells and neutrophils

B-cells and neutrophils have been less intensively studied, but the available data show that IL gene expression in these cells is also targeted by 1α,25(OH)_2_D_3_. In isolated human peripheral B-cells, IL-10 secretion can be induced by stimulation (cross-linking of B-cell receptor/CD40 antibody/IL-4). This production can be enhanced by 1α,25(OH)_2_D_3_. Besides the influence on IL gene expression, 1α,25(OH)_2_D_3_ induces the expression of VDR and Cyp24 mRNA in the stimulated B-cells. These activated cells also express Cyp27b1 mRNA and are able to produce 1α,25(OH)_2_D_3_ from 25(OH)D_3_. Binding of VDR to a VDRE in the proximal IL-10 promoter has been shown by ChIP assay, and binding of RNA-polymerase II could only be detected in IL-10 secreting B-cells (Heine et al., 2008).

Neutrophils respond to 1α,25(OH)_2_D_3_ by a slight reduction of IL-1β mRNA expression. Notably, the abundance of VDR mRNA in neutrophils has been found to be comparable with monocytes (Takahashi et al., 2002).

#### Fibroblasts, keratinocytes, endothelial cells

In a first study where these cell types were used, IL-1-stimulated normal human dermal fibroblasts, normal human keratinocytes and normal human endothelial cells were investigated regarding changes of IL-8 mRNA and protein expression in dependence of 1α,25(OH)_2_D_3_ treatment. IL-8 expression was reduced by 1α,25(OH)_2_D_3_ on both levels of gene expression for fibroblasts and keratinocytes, but not for endothelial cells, where no significant changes have been found (Larsen et al., 1991).

For IL-8, and also for IL-6 protein production, this result has been confirmed in studies using phorbol ester stimulated human fibroblast cell lines (Srviastava et al., 1994), and in experiments employing TNF-α-stimulated human dermal fibroblasts (Fukuoka et al., 1998). Similar results have been obtained with fibroblast cultures obtained from surgery of patients suffering from nasal polyposis, which is defined as a chronic inflammatory process. However, rather high concentrations (10–100 μM) of 1α,25(OH)_2_D_3_ were necessary to significantly reduce IL-6 and IL-8 production in these cells (Rostkowska-Nadolska et al., 2010).

In cultured normal human keratinocytes, only minor effects were observed for IL-1α and IL-8 production, when the influence of 1α,25(OH)_2_D_3_ was investigated for otherwise untreated cells. However, TNF-α-stimulation led to slightly enhanced IL-1α and markedly increased IL-8 secretion, which could be reduced by 1α,25(OH)_2_D_3_ (Zhang et al., 1994). This was confirmed for IL-8 (Koizumi et al., 1997). On the other hand, stimulation with phorbol ester plus LPS caused a rise in IL-8 production, but a decrease in IL-1α. 1α,25(OH)_2_D_3_ inhibited IL-8 secretion and restored IL-1α production (Zhang et al., 1994). Stimulation of normal human keratinocytes with IL-17A resulted in a pronounced increase in IL-6 mRNA and IL-8 protein secretion, which could be effectively blocked by 1α,25(OH)_2_D_3_ treatment (Peric et al., 2008). In a mechanistically insightful study, the effect of 1α,25(OH)_2_D_3_ on the expression of IL-1α, the intracellular IL-1 receptor antagonist (icIL-1Ra) and IL-18 was studied in mouse primary keratinocytes. Treatment with 1α,25(OH)_2_D_3_ induced IL-1α and icIL-1Ra mRNA and protein, however, the ratio of icIL-1Ra to IL-1, which determines the effect on IL-1 activity, was markedly increased, and indeed reduced IL-1 activity could be detected. The use of keratinocytes from VDR^−/−^ mice confirmed that the effect was mediated by VDR. Regarding the mechanism of gene regulation, increased IL-1α mRNA stability was observed and enhanced icIL-1Ra gene transcription via a secondary mechanism have been suggested to account for the effects on these gene. 1α,25(OH)_2_D_3_ markedly suppressed IL-18 mRNA expression, and the effect was dependent on VDR, as no effect of 1α,25(OH)_2_D_3_ was seen in VDR^−/−^ mice. These mice exhibit markedly elevated basal levels of IL-18 mRNA and protein, and expression of human VDR in these mice could restore basal levels (Kong et al., 2006).

A further cell type involved in inflammatory responses, especially in infection-mediated inflammation, are epithelial cells. Treatment of human microvessel endothelial cells with 1α,25(OH)_2_D_3_ suppresses LPS-induced IL-6 and IL-8 release, whereas 1α,25(OH)_2_D_3_ alone does not affect IL production. As assessed by reporter gene assay, this seems to be based on inhibition of LPS-induced NF-κB activation. This activation usually occurs via the MyD88-dependent branch of TLR4-signaling. In contrast, 1α,25(OH)_2_D_3_ did not influence the activity of interferon-β-promoter constructs, which has been determined as a measure of MyD88-independent LPS/TLR4 signaling (Equils et al., 2006). Reduced IL-6 and IL-8 production was also seen in 1α,25(OH)_2_D_3_-treated cystic fibrosis respiratory epithelial cell lines challenged with LPS. With respect to NF-κB-signaling, reduced IκBα phosphorylation and increased total cellular IκBα upon 1α,25(OH)_2_D_3_ treatment have been found in this study (McNally et al., 2011) (Figure 2). Similar findings have been made for human umbilical vein cord endothelial cells (HUVEC) incubated cultured in a CKD-like environment (hypocalcemia, advanced glycation end products, parathyroid hormone) and 1α,25(OH)_2_D_3_. This environment provoked enhanced IL-6 expression and secretion, increased DNA-binding of NF-κB-p65 and decreased IκBα expression. These changes were counteracted by 1α,25(OH)_2_D_3_ (Talmor-Barkan et al., 2011). In TNFα-stimulated human coronary arterial cells, a slight, but significant reduction of IL-8 production has been observed for 1α,25(OH)_2_D_3_ treatment in certain concentrations, but IL-6 production could not be influenced (Kudo et al., 2012). An interesting novel mechanism for interference of 1α,25(OH)_2_D_3_ and LPS-stimulated IL-8 production from epithelial cells has been proposed in a recent study, where a vitamin D_3_ derivative have been found to increase the release of the soluble form of CD14 (sCD14) via ERK1/2 activation. Neutralization of LPS by sCD14 could account for the effect of the vitamin D analog (Hidaka et al., 2013).

#### Trophoblasts, endometrial cells, myometrial cells

Placental inflammation including release of interleukins is associated with preeclampsia, preterm labor, and abortion. Therefore, cell types involved in this inflammatory condition have been investigated regarding the influence of 1α,25(OH)_2_D_3_ on IL secretion. In cultured human trophoblasts, 1α,25(OH)_2_D_3_ reduced TNFα-induced IL-6 mRNA expression and protein secretion (Diaz et al., 2009). Mechanistic evidence regarding the influence of vitamin D signaling on IL gene expression in placental tissue was presented in a study with for Cyp27b1^−/−^ (vitamin D-activating 1α-hydroxylase) mice and VDR^−/−^ mice. In these mice, basal expression of IL-10 mRNA was decreased relative to wildtype placentas, and LPS stimulation resulted in higher levels of IL-6 mRNA in the ^−/−^ placentas compared to wildtype. PCR array analysis of LPS-stimulated placental tissue from Cyp27b1^−/−^ mice revealed enhanced expression of IL-4, IL-15, and IL-18 mRNA relative to WT and the same experiments with VDR^−/−^ mice yielded higher IL-1α and IL-6 mRNA levels. Further experiments with LPS-stimulated placentas from WT mice showed that treatment with 25(OH)D_3_ as the substrate of CYP27B1 reduces IL-6 mRNA expression. Moreover, LPS challenge of pregnant WT mice led to enhanced expression of Cyp27b1 and VDR. Apart from the mechanistic conclusion that VDR signaling is a factor that controls IL gene expression, these results show that pro-inflammatory stimuli are able to enhance the expression of crucial vitamin D signaling components which are able to mediate anti-inflammatory responses (Liu et al., 2011).

In line with these findings, experiments using human endometrial cells from women with unexplained recurrent spontaneous abortion (URSA) or in controls, significant down-regulation of IL-6 by 1,25(OH)_2_D_3_ was observed in two cell types (whole endometrial cells and endometrial stromal cells), but for IL-8, opposed effects were observed for the two cell types in URSA samples, which highlights the complexity of these responses given the fact that several cell types are involved in inflammatory processes (Tavakoli et al., 2011).

#### Adipocytes

Obesity is a disease condition which is strongly associated with low-grade inflammation, therefore adipocytes have been used as a further model system regarding the interplay of vitamin D signaling and IL gene expression/production. In a recent report, human adipocytes from biopsies and from differentiated human mesenchymal stromal cells were studied with respect to IL-6 gene expression/release depending on the presence of 1α,25(OH)_2_D_3_. LPS-induced IL-6 mRNA and protein were reduced in both systems by cotreatment with 1α,25(OH)_2_D_3_. Regarding the underlying signal transduction events, it was shown that 1α,25(OH)_2_D_3_ inhibited IκB phosphorylation and thus NF-κB translocation into the nucleus (Figure 2). DNA binding of NF-κB complexes upon LPS stimulation was significantly reduced in 1α,25(OH)_2_D_3_-pretreated cells compared to controls (Mutt et al., 2012). A further recent investigation addressed the influence of *in vitro* and *in vivo* administered 1α,25(OH)_2_D_3_ on IL-6 and IL-8 gene expression from IL-1β-stimulated human adipose tissue. The adipose tissue samples have been either (i) treated *in vitro* with 1α,25(OH)_2_D_3_ or have been (ii) obtained from obese subjects with low plasma levels of 25(OH)D_3_ after *in vivo* (oral) treatment with high-dose 1α,25(OH)_2_D_3_ or placebo. In the *in vitro* study, reduced mRNA levels of IL-6 and IL-8 and reduced IL-6 and IL-8 protein (significance only shown for IL-8) have been found. However, although the *in vivo* treatment led to a small decrease of IL-6 and IL-8 mRNA expression in the adipose tissue, there were no significant differences between the 1α,25(OH)_2_D_3_–treated and the control group. Oral treatment with 1α,25(OH)_2_D_3_ did also not significantly change circulating levels of IL protein in the subjects pre- and post-treatment (Wamberg et al., 2013). These findings urge caution about the extrapolation of *in vitro* findings to the *in vivo* situation.

Apart from studies with primary cells, cultures of adipocyte-like murine 3T3-L1 cells have been used, but contradictory results have been reported e.g., regarding IL-6 gene expression (Sun and Zemel, 2008; Marcotorchino et al., 2012).

### VDR gene variants, VDR gene silencing, and IL gene expression/production

A further aspect that underscores the importance of vitamin D signal transduction on IL biosynthesis is the effect of the VDR receptor gene variants on IL gene expression. The single-nucleotide polymorphism *Fok*I, which comprises a shorter VDR protein of 424 aa or the long isoform with 427 aa, influences IL-12 expression. In human monocytes and DCs, presence of the short VDR isoform leads to a higher expression of IL-12 compared to the long isoform, a result which was reflected by results from reporter gene assays with IL-12 promoter fragments (Van Etten et al., 2007). Moreover, VDR gene promoter variants have an impact on the expression of IL-10 in blood mononuclear cells (Selvaraj et al., 2008).

Changes in IL production can be observed in VDR KO mice. VDR KO considerably facilitates development of IL-17 secreting T-cells (T_h_17 cells) in response to respective *in vitro* stimuli. Further, enhanced IL-17 production was observed in these T_h_17 cells compared to wildtype. Conversely, a reduction in regulatory T-cells and tolerogenic DCs was observed. Moreover, IBD can be induced experimentally in these mice by transfer of naive T-cells that develop into specific, IBD-inducing subsets. The severity of IBD was strongly enhanced in VDR KO mice compared to control animals, which was ascribed to the increased propensity for development into T_h_17 cells (Bruce et al., 2011).

### Influence of 1α,25(OH)_2_D_3_ on IL receptor expression

Apart from induction of IL gene expression/protein release, 1α,25(OH)_2_D_3_ may also modulate IL signaling via regulation of IL receptor expression. In early reports, moderate downregulation (Matsui et al., 1986) or no changes (Jordan et al., 1989) were found regarding IL-2 receptor expression in 1α,25(OH)_2_D_3_ treated, mitogen-stimulated PBMC, or mitogen/phorbol ester-stimulated T-cells, respectively. However, IL-2 mediated expression of IL-2 receptor units was superinduced by 1α,25(OH)_2_D_3_ in mitogen-stimulated PBMC (Rigby et al., 1990). The vitamin D_3_ upregulated protein 1 (VDUP1), which is expressed in a 1α,25(OH)_2_D_3_-dependent manner, has been found to inhibit the activity of the IL-3 receptor promoter (Han et al., 2003). On the other hand, IL-1 and IL-4 receptor densities seem to be upregulated by 1α,25(OH)_2_D_3_ on a murine T-cell line and a murine osteoblast cell line, respectively (Lacey et al., 1993a,b). Furthermore, downregulated IL-22 mRNA and protein levels have been detected in cultured epidermis tissue treated with calcipotriol, a vitamin D analog (Moniaga et al., 2013).

### The influence of 1α,25(OH)_2_D_3_ on TNFα mRNA and protein expression and release

The impact of 1α,25(OH)_2_D_3_ on TNFα gene expression was primarily studied in PBMC, primary monocytes/macrophages or in monocytic cell lines. Expression was investigated both on mRNA level and/or on the level of protein production, and was sometimes reported in terms of protein release as a secondary readout.

In general, *in vitro* or *in vivo* treatment with 1α,25(OH)_2_D_3_ of PBMC caused a decrease in TNFα gene expression and/or TNFα production. This was the case for PBMC from healthy donors that were stimulated with different agents (LPS, Muller et al., 1992; Panichi et al., 1998; Rausch-Fan et al., 2002); live *Mycobacterium tuberculosis* (Prabhu Anand et al., 2009), as well as for PBMC from patients suffering from diseases with inflammatory features [renal disease (Riancho et al., 1993; Panichi et al., 1998); pulmonary tuberculosis (Prabhu Anand et al., 2009)]. Analogous findings were obtained with monocyte-enriched PBMC after stimulation with LPS (Muller et al., 1992), IFNγ or phorbol ester (Zarrabeitia et al., 1992) (however, not with LPS in this particular report). In one of the latter studies, nuclear run-off analysis did not indicate that TNFα gene transcription was affected by 1α,25(OH)_2_D_3_ (Muller et al., 1992).

In contrast to the findings with PBMC or monocyte-enriched PBMC, studies that used human primary monocytes or macrophages often found increased TNFα expression/secretion after 1α,25(OH)_2_D_3_ exposure, either regarding basal levels (human monocyte-derived macrophages; Bermudez et al., 1990) or with respect to stimulus-induced mRNA or protein levels (murine alveolar macrophages/LPS- or PMA-stimulation, Higashimoto et al., 1995; peritoneal macrophages from continuous peritoneal dialysis patients/LPS-stimulation, Cohen et al., 2001). In line with this, murine bone-marrow derived macrophages (BMMs) responded to 1α,25(OH)_2_D_3_ with an increase in TNFα mRNA abundance, which was synergistically enhanced by LPS stimulation. This study also addressed molecular mechanisms. Treatment with 1α,25(OH)_2_D_3_ and stimulation with LPS did not influence TNFα mRNA stability, but the data suggested that 1α,25(OH)_2_D_3_ regulates the TNFα gene on transcriptional level, as a VDR-binding sequence could be identified in the TNFα promoter region using electrophoretic mobility shift assays (Hakim and Bar-Shavit, 2003).

When human monocytic cell lines were studied, heterogeneous results were obtained, and the outcome seems to depend on the differentiation status of the cells (e.g., Bhalla et al., 1991). For the three cell lines that were mainly employed, the order of their stage of maturation is known. HL-60 cells are myelomonocytic stem-cells and thus are the least mature cell line; U937 are characterized as monoblasts, and represent an intermediate stage; and THP-1 cells are regarded as promonocytic cells and are therefore the most mature cell line (Frankenberger et al., 1994).

In HL-60 cells, 1α,25(OH)_2_D_3_ had no influence on PMA-induced TNFα mRNA expression, but enhanced it in U937 cells (Bhalla et al., 1991). In a second study, 1α,25(OH)_2_D_3_ preincubation of U937 cells accelerated LPS-induced TNFα mRNA expression and led to higher steady-state mRNA levels which were associated with enhanced TNFα protein production. Mechanistic analysis pointed to a secondary effect since 1α,25(OH)_2_D_3_ pretreatment was needed for more than 6 h in order to achieve enhanced TNFα protein synthesis. The requirement of 1α,25(OH)_2_D_3_-driven expression of the LPS co-receptor CD14, was suggested to be the mechanistic basis of his secondary effect (Prehn et al., 1992). In a further investigation, differentiation by 1α,25(OH)_2_D_3_ enhanced LPS-induced TNFα secretion in U937 and THP-1 cells. Concomitant increase in TNFα mRNA was confirmed for U937 cells (Taimi et al., 1993). In contrast, 1α,25(OH)_2_D_3_ was reported to significantly suppress TNFα release in LPS-stimulated THP-1 cells and human primary monocytes (Kuo et al., 2010), and a further study reported reduced TNFα production and secretion from 1α,25(OH)_2_D_3_-treated, IFNγ-activated THP-1 cells (Villaggio et al., 2012).

In one report, TNFα mRNA levels of 1α,25(OH)_2_D_3_–treated human PBMC, U937 and THP-1 cells, that were stimulated either with LPS or with phytohemagglutinin (PHA), were compared. Differences occurred between the two sample types and the two stimuli. In PBMC, LPS had no influence on TNFα expression in the presence of 1α,25(OH)_2_D_3_, whereas upon PHA-stimulation, reduced TNFα mRNA levels were observed. In contrast, U937 cells (but not THP-1 cells) responded by an increase in TNFα mRNA expression (Blifeld et al., 1991).

Taken together, several studies report an increase in TNFα mRNA and protein expression in 1α,25(OH)_2_D_3_-treated, subsequently stimulated U937 cells, but equivocal effects were found with the more mature THP-1 cells. In monocyte-derived DCs from patients that suffer from Crohn's disease, TNFα production was decreased when the cells were differentiated with LPS in the presence of 1α,25(OH)_2_D_3_ (Bartels et al., 2013).

T-cells have not been intensively studied, but regulation of TNFα-expression by 1α,25(OH)_2_D_3_ has been analyzed in T-cell subsets obtained from normal healthy subjects and pulmonary tuberculosis patients. Here, 1α,25(OH)_2_D_3_ reduced the percentage of TNFα-expressing T-cell subsets (CD3+, CD3+CD4+, CD3+CD8+) (Prabhu Anand et al., 2009) (Figure 3).

Other cell types that were analyzed are prostate cancer lines, where 1α,25(OH)_2_D_3_ reduced basal TNFα mRNA expression (Golovko et al., 2005), or 1α,25(OH)_2_D_3_/IL-1β-stimulated synoviocytes, where TNFα mRNA was decreased (Feng et al., 2013).

In summary, 1α,25(OH)_2_D_3_-mediated downregulation of TNFα gene expression has been found in cell preparations which contain a high percentage of T-cells (PBMC or monocyte-enriched PBMC). In monocytic cells, upregulation has been reported for cell lines that represent an intermediate monocytic differentiation state, whereas for more mature cells, heterogeneous results have been found. Regarding the mechanism, it has been suggested that primary effects may play a role for 1α,25(OH)_2_D_3_ regulation of TNFα gene expression, since a VDR binding element has been found in the TNFα promoter region (Hakim and Bar-Shavit, 2003). On the other hand, kinetic analysis pointed to a secondary effect, where the expression of CD14 could play a role, at least for LPS-induced TNFα expression (Prehn et al., 1992). It has to be noted, however, that cell-type specific mechanisms have been found for T-cell specific expression of the TNFα gene. Cell type-specific DNA-protein-interactions have been identified for the TNFα gene when T-cells and monocytic cells were compared. A highly conserved region in intron 3 seems to be responsible for cell specificity, as this sequence induces specific activity of a TNFα-reporter plasmid in Jurkat T-cells, but not THP-1 cells (Barthel and Goldfeld, 2003). Possibly, cell specific protein complexes within this region interact with 1α,25(OH)_2_D_3_ signaling components in T-cells.

### The influence of 1α,25(OH)_2_D_3_ on interferon γ gene expression

IFNγ is a well-established effector in anti-infectious host reactions, autoimmune diseases and inflammation. IFNγ is mainly produced by NK and T-cells. Inhibition of IFNγ mRNA and protein secretion has been described for 1α,25(OH)_2_D_3_-treated human PBMC, peripheral blood lymphocytes or T-cells that were stimulated with phytohemagglutinin and phorbol ester (Matsui et al., 1986; Reichel et al., 1987; Rigby et al., 1987; Inoue et al., 1998) (Figure 3). Mechanistic insights exist from experiments using transient transfection of IFNγ promoter constructs in Jurkat T-cells. Here, it could be concluded that two VDR binding regions, one around −200 bp from the transcription start site and the second directly around the transcription start site, are involved in the regulation of IFNγ gene expression by 1α,25(OH)_2_D_3_ (Cippitelli and Santoni, 1998).

## Conclusions

It is well established that 1α,25(OH)_2_D_3_ influences cytokine gene expression and signaling in several different cell types. Firstly, this is the case for the pleiotropic mediator TGF-β, for which it has been shown that either the expression of the cytokine itself or expression of associated signaling components is downregulated by 1α,25(OH)_2_D_3_. In hepatocytes, 1α,25(OH)_2_D_3_ has been found to influence TGF-β signaling in a genome wide scale by directing binding of Smad proteins to target genes. These actions of 1α,25(OH)_2_D_3_ on TGF-β expression or signaling were able to inhibit fibrosis and associated inflammation. Second, the interleukins are a vast group of inflammatory cytokines that are clearly regulated by 1α,25(OH)_2_D_3_ in a cell-specific manner. However, for several members of this family (e.g., IL-1, IL-6, and IL-8), both positive or negative regulation by 1α,25(OH)_2_D_3_ has been observed. A closer look at the parameters that determine the outcome of 1α,25(OH)_2_D_3_ action on the expression of these genes is warranted. This applies in particular to the time-scale of changes in gene expression, as different responses may occur during separate stages of 1α,25(OH)_2_D_3_ action. Regarding the mechanisms, recruitment of VDR to the respective genomic regions, as well as interaction of 1α,25(OH)_2_D_3_ signaling with other transcription factors involved in IL expression (NFAT, NF-κB, Runx1), seem to occur. Concerning the p38 MAP kinase phosphatase MKP1, it was found that GCR and VDR/RXR act in a synergistic manner to induce MKP1 expression in monocytes. This results in reduced p38 activation and reduced formation of proinflammatory cytokines. As a further cytokine, the proinflammatory mediator TNFα has been identified as a 1α,25(OH)_2_D_3_ target gene. Also in this case, the vitamin D effects are cell-specific: With cell samples that mainly contain T-cells, downregulation of TNFα has been observed, whereas for monocytic cells, either positive or negative regulation occurred depending on the differentiation state. Finally, gene expression of the proinflammatory mediator IFNγ has been described to be suppressed by 1α,25(OH)_2_D_3_ in T-cells. Altogether, the influence of 1α,25(OH)_2_D_3_ on the expression of interleukins, TNFα, and IFNγ by different cell types, and the consequences for the cellular interplay that are to be anticipated, amounts to a complex picture. In Figure 3, the influence of 1α,25(OH)_2_D_3_ on the expression of these cytokines is summarized for the major immune cells (monocytes, DCs, and different T-cell subsets). The resulting pattern supports a shift of T-cell responses from a Th1 type toward Th2 reactions and a suppression of Th17 responses. The effect of 1α,25(OH)_2_D_3_ on cytokine expression in antigen presenting cells (monocytes, DCs) remains unclear and seems to depend on the time of stimulation, the differentiation state and other factors.

## Perspectives

Modulation of GCR, NFκB, NFAT as well as SMAD signaling plays a central role in the immunomodulatory activities of 1α,25(OH)_2_D_3_. Mechanistic studies on individual genes gave some mechanistic insights into the mechanisms involved in the interaction between VDR/RXR and the above mentioned transcription factors. These mechanisms include competitive binding as well as a crosstalk between the signaling pathways on multiple levels including the promoter level. However, by using ChIP seq and other techniques which allow a genome-wide view, we are just starting to understand the signaling network which is responsible for cell-type-specific and locus-dependent gene activation by ligand-regulated transcription factors such as VDR/RXR. For example, intersecting VDR/SMAD regulatory circuits have just been unraveled and it was shown that TGFβ signaling facilitates VDR binding to certain gene loci. More such data are required to increase our understanding of the complex gene regulatory network that is affected by 1α,25(OH)_2_D_3_. Especially, genome-wide data on VDR loci in conjunction with analyses of other, inflammation-related key transcription factors in different cell types and various stimuli are necessary to understand the complex regulation of gene transcription during inflammation.

### Conflict of interest statement

The authors declare that the research was conducted in the absence of any commercial or financial relationships that could be construed as a potential conflict of interest.
